# Exploring the Interaction Natures in Plutonyl (VI) Complexes with Topological Analyses of Electron Density

**DOI:** 10.3390/ijms17040414

**Published:** 2016-04-11

**Authors:** Jiguang Du, Xiyuan Sun, Gang Jiang

**Affiliations:** 1 College of Physical Science and Technology, Sichuan University, Chengdu 610064, China; 2 College of Sciences, Sichuan Agricultural University, Ya′an 625014, China; sunxy@sicau.edu.cn; 3 Institutes of Atomic and Molecular Physics, Sichuan University, Chengdu 610065, China; gjiang@scu.edu.cn

**Keywords:** plutonyl complexes, chemical bondings, QTAIM, NCI

## Abstract

The interaction natures between Pu and different ligands in several plutonyl (VI) complexes are investigated by performing topological analyses of electron density. The geometrical structures in both gaseous and aqueous phases are obtained with B3LYP functional, and are generally in agreement with available theoretical and experimental results when combined with all-electron segmented all-electron relativistic contracted (SARC) basis set. The Pu–$O_{yl}$ bond orders show significant linear dependence on bond length and the charge of oxygen atoms in plutonyl moiety. The closed-shell interactions were identified for Pu-Ligand bonds in most complexes with quantum theory of atoms in molecules (QTAIM) analyses. Meanwhile, we found that some Pu–Ligand bonds, like Pu–OH^−^, show weak covalent. The interactive nature of Pu–ligand bonds were revealed based on the interaction quantum atom (IQA) energy decomposition approach, and our results indicate that all Pu–Ligand interactions is dominated by the electrostatic attraction interaction as expected. Meanwhile it is also important to note that the quantum mechanical exchange-correlation contributions can not be ignored. By means of the non-covalent interaction (NCI) approach it has been found that some weak and repulsion interactions existed in plutonyl(VI) complexes, which can not be distinguished by QTAIM, can be successfully identified.

## 1. Introduction

Plutonium is the only element in the periodic table that can have appreciable amounts of four different oxidation states existing in aqueous acidic solutions simultaneously. Therefore, the structures, redox, and interactive features of plutonium complexes are needed to be investigated by using experimental or theoretical methods. Plutonium complexes were significantly less studied in experiments relative to its thorium and uranium counterparts for reasons of toxicity and radioactivity. Raman spectra of plutonyl (VI) ions in solution were measured by Madic *et al.* [1] to identify the vibrational transition.Pu–L_3_ X-ray near edge absorption spectra for Pu (0, VII) were measured by Conradson *et al.* [2] for a large number of compounds. A number of novel and unexpected behaviors were observed in their experiment. The chloride plutonyl (VI) complexes were investigated in NaCl solutions using conventional absorption spectrophotometry [3]. The experiment suggests that mixed chloro-hydroxo or chloro-carbonate may form at high pH due to strong affinity of plutonyl (VI) for chloride. Recently, the crystal structure of the plutonyl (VI) dinitrate complex was characterized with spectroscopic methods by Gaunt *et al.* [4]. They found the dominant formation of mononitrate and negligible formation of the dinitrate in aqueous solutions. In contrast to experiments, tremendous theoretical works with quantum mechanical methods have been performed to probe the structural and electronic properties and spectra of plutonyl complexes. There are a number of theoretical data on plutonyl aquo [5,6,7], carbonate [8], hydroxo [9] and fluoride [10,11] complexes. Odoh *et al.* [12] have recently utilized DFT methods to investigate the structural properties of various Pu (V) and Pu (VI) complexes. This and other previous reports would suggest that Kohn–Sham DFT can be successfully used in predicting the structures of Pu (IV) and Pu (VI) complexes. The chemical bonding natures of Uranyl (VI) complexes have been recently identified by Vallet *et al.* [13] with electron density topological analyses methods, namely the quantum theory of atoms in molecules (QTAIM) proposed by Bader [14]. Kaltsoyannisaŕ group [15] also performed topological analyses for actinides compounds. These reports significantly indicate that QTAIM is an appropriate and successful tool to access the bonding characters of actinides complexes.

In this paper, we first determined the structures of various plutonyl (VI) complexes with OH^−^, Cl^−^, (F^−^/Br^−^, $O_{2}^{2-}$, ${\text{NO}}_{3}^{-}$, ${\text{CO}}_{3}^{2-}$, H_2_O ligands in gaseous and aqueous phases within DFT framework. Based on the gaseous electron density, the QTAIM and interaction quantum atom (IQA) [16] analyses were performed to identify the interaction nature between Pu and coordinating ligands. In addition, another density-based analytical tool, the electron localization function (ELF) [17], was utilized to study the chemical bondings. As an excellent extension of QTAIM theory, the non-covalent interaction (NCI) index proposed by Yang *et al.* [18] was used to probe into the weak interactions in the complexes studied.

## 2. Computational Details

Calculations were performed with two different packages, ORCA-3.0.0 [19] and G09 [20]. All calculations in gaseous and aqueous phases were done at DFT level of theory with functional forms of B3LYP [21]. The geometry structures of titled complexes in gaseous and aqueous phases were optimized without symmetry constrain, and were identified to be one minimum in potential energy surface through vibrational frequency calculations. All solution phase calculations were carried out in water.

In ORCA calculations, the scalar relativistic effect was evaluated with zero-order regular approximation (ZORA) [22]. Segmented all-electron relativistic contracted (SARC) Gaussian-type basis sets [24] are assigned to Pu atom for optimization, and other atoms in the studied complexes were considered with all-electron TZV-ZORA basis set [23]. The conductor-like screening model (COSMO) [25] is utilized to evaluate the solvent effect. In G09 calculations, the B3LYP functional in conjunction with relativistic effective core pseudopotential (RECP) was utilized for optimization, the Stuttgart small-core relativistic effective core potential (RECP) with 60 core electrons frozen and assisted basis set [26,27] were employed to describe Pu atom, and aug-cc-PVDZ basis set [28] for light elements. The ultrafine grid (99, 590) was used in the integral calculations to accurately describe heavy element Pu. The solution phase calculations in G09 were carried out with the polarizable continuum model (PCM) [29]. The calculational scheme with G09 is defined as B3LYP/SDD in the following.

The QTAIM, IQA, energy decomposition scheme and NCI analyses were performed to deeply understand the interaction natures between Pu and different ligands in studied plutonyl (VI) complexes. The wave-function used in these bonding analyses were obtained at the B3LYP level of theory. The QTAIM and IQA calculations were carried out with AIMAll [30], and NCI with Multiwfn [31] programs.

## 3. Results and Discussion

### 3.1. Structures in Gaseous and Solution Phases

We first optimized the structures of bare plutonyl moieties (${\text{PuO}}_{2}^{2/1+}$) and pentaaquo complexes to test the reliability of DFT functionals and basis set employed in our present work. The calculated structural parameters are listed in Table 1. Our results indicate that that the Pu–$O_{yl}$ bond lengths calculated with B3LYP/SARC-ZORA are closer to experimental values [1,32] relative to the B3LYP/SDD results. Our B3LYP results are in agreement with other theoretical values obtained at *ab initio* [33,34] and DFT levels [12]. The consideration of solution effect results in the elongation of Pu–$O_{yl}$ bonds and the shortening of Pu–OH_2_ bonds for all studied aquo complexes. Similar effects have been observed in recent DFT calculations [12]. It is worth noting that the Pu–$O_{yl}$ and Pu–OH_2_ bond lengths are closer to the experimental values when the solution effect is considered.

**Figure 1:**
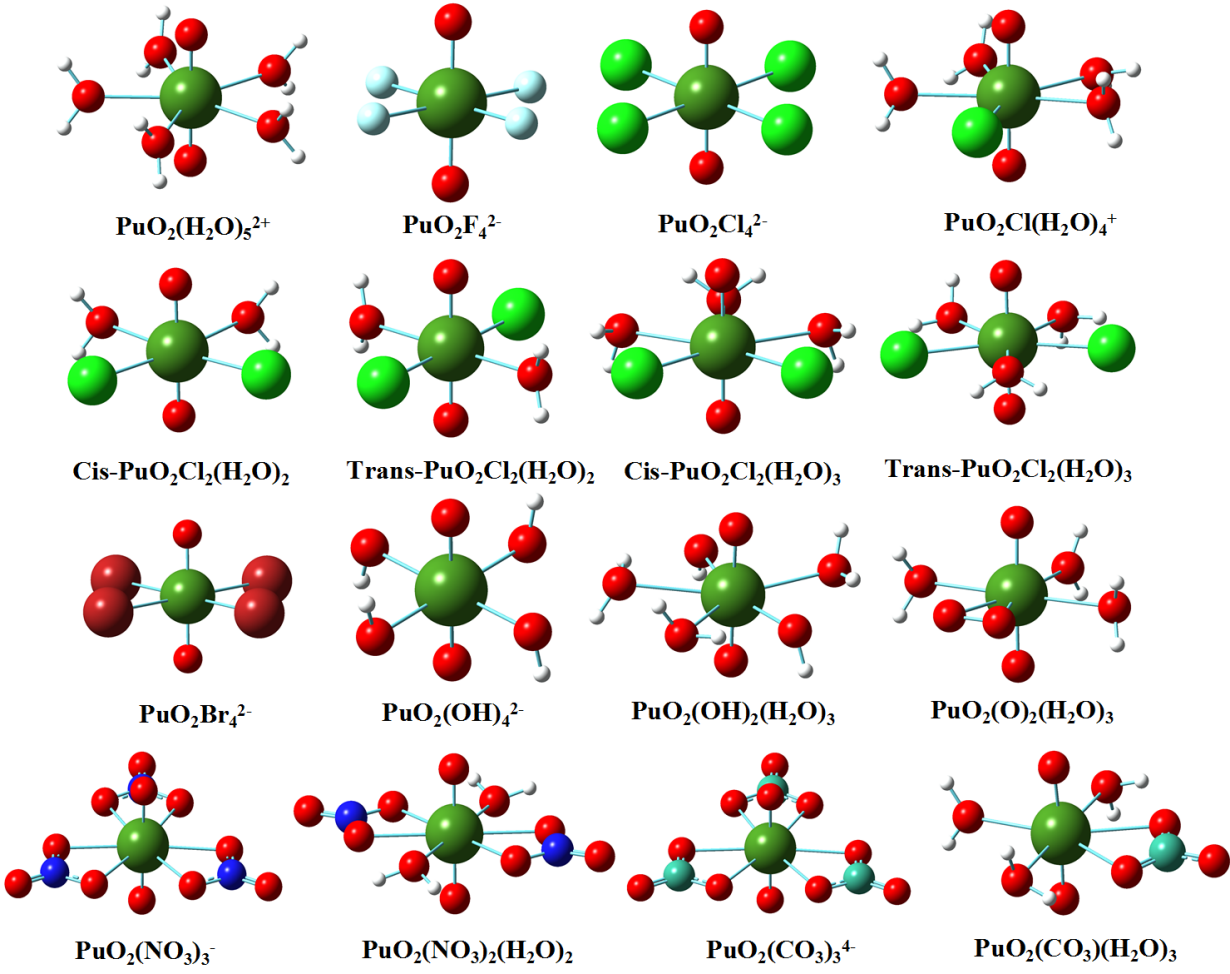
The relaxed geometry structures of Plutonyl (VI) complexes in gas phase optimized at B3LYP/SARC-ZORA level of theory.

The geometrical structures of the plutonyl (VI) complexes with ligands, F^−^, Cl^−^, Br^−^, OH^−^, ${\text{NO}}_{3}^{-}$, $O_{2}^{2-}$ and ${\text{CO}}_{3}^{2-}$ obtained at the B3LYP/SARC-ZORA level of theory are depicted in Figure 1. The typical bond lengths calculated with B3LYP/SARC-ZORA and B3LYP/SDD, respectively, are collected in Table 2 for the sake of comparison. On the whole, the Pu–$O_{yl}$ bond lengths calculated with solution effect considered are slightly larger than those obtained in gas-phase calculation for all studied complexes. On the other hand, the consideration of solution results in contraction of the Pu–OH_2_ bond lengths and elongation of of Pu–Ligand bonds. In PuO_2_$F_{4}^{2-}$, PuO_2_${\text{Cl}}_{4}^{2-}$, PuO_2_${\text{Br}}_{4}^{2-}$, PuO_2_${\left(\text{OH}\right)}_{4}^{2-}$ complexes, the Pu–L bond lengths show the order of Pu–F<Pu–OH<Pu–Cl<Pu–Br, and the Pu–F, Pu–Cl, Pu–Br and Pu–OH bond lengths in aqueous phase were calculated as 2.201, 2.706, 2.853 and 2.243 Å, respectively, when the B3LYP/SARC-ZORA was employed. We also investigated the geometry properties of mono- and bis-chloro complexes which have been identified in NaCl solutions by Runde *et al.* [3]. Energetically, two isomers of PuO_2_Cl_2_(H_2_O)_2_ are very close to each other, with the difference of 4.82, 0.19 kcal/mol, respectively, in gas-phase and aqueous phase at B3LYP/SARC-ZORA level of theory. Both isomers show the similar structural features as shown in Table 2. The trans-isomer of PuO_2_Cl_2_(H_2_O)_3_ complexes is slightly more stable than cis-isomer by 8.32 kcal/mol (gas), 2.11 kcal/mol (aquo), respectively. There also exists no great difference in geometry parameters for both isomers. It should be noted that the structural parameters of PuO_2_Cl_2_(H_2_O)_3_ in aqueous phase with B3LYP/SARC-ZORA are excellent in agreement with available experimental values [3]. The mono-chloro complex (PuO_2_Cl(H_2_${O)}_{4}^{+}$) possesses shorter Pu–$O_{yl}$ and Pu–OH_2_ bonds relative to bis-chloro complexes. This result agrees well with previous experimental [3] and theoretical investigations [12]. The distance between Pu and Cl atoms in PuO_2_Cl(H_2_${O)}_{4}^{+}$ is 2.722 Å at B3LYP/SARC-ZORA level, larger than previous DFT calculations [12] by 0.08 Å, is however closer to the experimental value of 2.75 Å. In both gaseous and aqueous phases, the bond lengths of Pu–$O_{yl}$ and Pu–OH^−^ in hydroxide complex, PuO_2_(OH)_2_(H_2_O)_3_, are significantly shorter than that in PuO_2_(OH${)}_{4}^{2-}$. For PuO_2_(O)_2_(H_2_O)_3_ complex, the distance between Pu and coordinating oxygen atom of $O_{2}^{2-}$ ligand is longer than Pu–OH^−^ bond length in PuO_2_(OH)_2_(H_2_O)_3_ complex. It should be mentioned that the U–OH^−^ bond is shorter than U–$O_{2}^{2-}$ bond in respective triaquo U(VI) analogies. This indicates that plutonyl (VI) shows weak affinity to $O_{2}^{2-}$ ligand relative to plutonyl (VI). In experiments, Gaunt *et al.* [4] recently characterized the structure of Plutonyl (VI) diaquo and dinitrate. The Pu–$O_{yl}$, Pu–$O_{L}$ (L = ${\text{NO}}_{3}^{-}$) and Pu–OH_2_ bonds lengths were calculated with B3LYP functional as 1.767, 2.483 and 2.466 Å, respectively, and are excellent in agreement with experimental measures [4] shown in Table 2. The typical bonds in trinitrate complex are not significantly different from that in dinitrate complex. For plutonyl (VI) carbonate complexes, the Pu–$O_{yl}$, Pu–$O_{L}$ (L = ${\text{CO}}_{3}^{2-}$) and Pu–OH_2_ bond lengths are all larger than the counterparts in plutonyl (VI) nitrate complexes.

### 3.2. Natural Bonding Orbital Analyses

**Figure 2:**
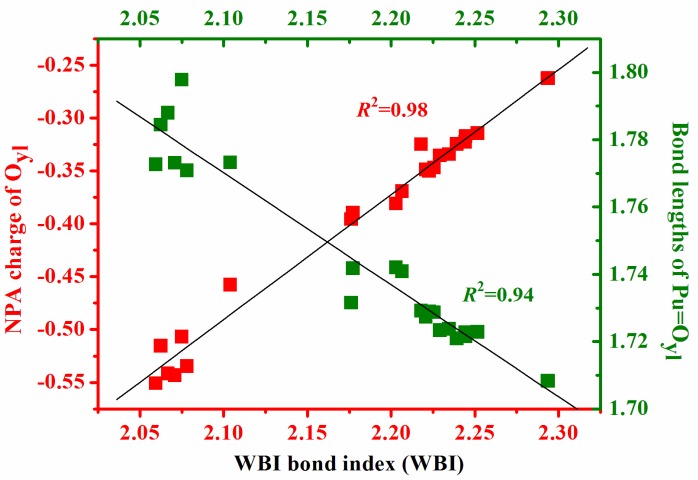
The linear correlation between WBI values and Pu–O_*yl*_ bond lengths and NPA charge of O_*yl*_ atoms in plutonyl moetiy.

The charge (*q*) based on natural population analysis (NPA) [33] and electronic configurations (EC) of Plutonium, Oxygen in plutonyl ($O_{yl}$) and coordinating atoms in ligands (L) of studied Plutonyl (VI) complexes are summarized in Table 3. The Pu atoms with about 1e lost act as electron donor in all complexes as expected. One can find from Table 3 that the Pu atoms possess $5f^{5}6d^{1.5}7p^{0.5}$-like electron configuration. Comparing with the population of free Pu atom, $5f^{6}7s^{2}$, there are significant electron promotions from 7s orbital to 6d and 7p orbitals when Pu atoms are involved in chemical bonds. On the other hand, the oxygen atoms in plutonyl moiety and coordinating atoms in studied Lewis acid ligands accept electrons from Pu atoms. The Wiberg bond index (WBI) in Table 3 indicates that the Pu-$O_{yl}$ bonds in studied complexes have double bond features. Other chemical bonds—Pu–F, Pu–Cl, Pu–Br and Pu–OH—show single bond nature. In the peroxide, nitrate and carbontrate complexes, the bond orders between Pu and coordinating oxygen are significantly smaller than other Pu–L bonds. We also found that the interaction between Pu and H_2_O ligand is very weak from WBI values. The WBI values of Pu–$O_{yl}$ bonds *vs.* corresponding bond lengths and charge of oxygen atoms in plutonyl moiety are depicted in Figure 2, from which one can find that there is good linear correlation with $R^{2}=0.94$ between Pu–$O_{yl}$ bond lengths and corresponding WBI values. This can be easily understood that the bigger the bond orders, the shorter the Pu–$O_{yl}$ bond lengths. It is very interesting that the Pu–$O_{yl}$ bond orders also show perfect linear dependence on the charge of oxygen atoms in plutonyl moiety ($R^{2}=0.98$). The oxygen atoms in Pu–$O_{yl}$ chemical bonds which accept fewer electrons generally possess the larger bond orders. Therefore, the Pu–$O_{yl}$ chemical bonds are not mainly derived from charge transfer mechanism. This implies that the interactive nature between Pu and $O_{yl}$ is covalent predominantly, not ionic features.

### 3.3. QTAIM Topological Analyses

**Figure 3:**
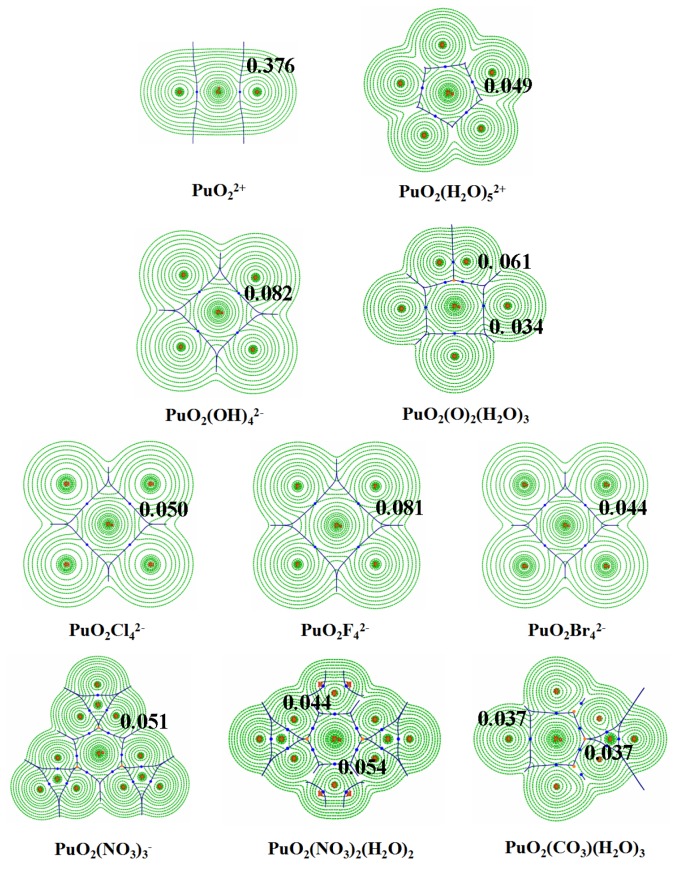
Electron density in the equatorial plane of studied plutonyl (VI) complexes, the BCPs are shown as the small blue sphere; the thin green lines represent the counter map of density; thin blue lines represent the interatomic paths that separate the atom electron density basins; the values of the density at the BCPs are also shown.

The wavefunction obtained at B3LYP level of theory is used to analyze the electron densities and further topological properties within QTAIM framework for Plutonyl (VI) species studied. The gas-phase electron densities corresponding to (3, −1) bonding critical points (BCPs) in the equatorial plane of the studied complexes are depicted in Figure 3. In bare ${\text{PuO}}_{2}^{2+}$ moiety, there is significant electron accumulation between Pu and O atoms, consistent with WBI results mentioned above. However, there is practically no built-up of electron density between Pu and H_2_O/${\text{CO}}_{3}^{2-}$/$O_{2}^{2-}$/${\text{NO}}_{3}^{-}$, implying the closed-shell interaction characteristics. It should be mentioned that previous works found significant electron density accumulation between Uranium and coordinating oxygen atoms in $O_{2}^{2-}$ and ${\text{CO}}_{3}^{2-}$ ligands. However, Plutonyl peroxide and carbonate complexes do not show similar interaction feature to Uranyl analogies. [13]. For PuO_2_$F_{4}^{2-}$, PuO_2_${\text{Cl}}_{4}^{2-}$, PuO_2_${\text{Br}}_{4}^{2-}$, PuO_2_(OH${)}_{4}^{2-}$ complexes, the minor electron accumulations is presented between Plutonium and F^−^/Cl^−^/Br^−^/OH^−^.

To deeply understand the interactive natures between Pu and coordinating atoms in complexes studied, the electron density (*ρ*) at (3, −1) BCPs, Laplacian of electron density ($\nabla^{2}\rho$), energy density H(r), delocalization indices δ(A,B) and −V(r)/G(r) ratio are collected in Table 4. According to QTAIM theory, the covalent interaction has a negative $\nabla^{2}\rho$ at the critical point (CP). However, this criterion has been proved to be not sufficient to describe the bond natures of heavy atoms in previous work [36]. Another property, the total energy density H(r) (defined as the sum of local kinetic energy density G(r) and the local potential energy density V(r)) proposed by Cremer [37] was proved to be very appropriate to characterize the degree of covalency of a bond, the more negative the H(r) value, the more stabilizing the interaction. One can also employ the −V(r)/G(r) ratio as another useful description, the −V(r)/G(r)<1 is characteristic of a typical ionic bond; and −V(r)/G(r)>2 is diagnostic of a classical covalent interaction. As shown in Table 4, the $\nabla^{2}\rho$ values are positive for all studied Pu-L BCPs, and show the sign of closed-shell interaction nature. The strong Pu–$O_{yl}$ covalent interaction is clearly revealed by great negative H(r) value, large *δ*(Pu, L) and −V(r)/G(r) ratio being close to 2. The positive $\nabla^{2}\rho$ and H(r) values of Pu–OH_2_ BCPs in all complexes studied strongly suggest closed-shell interactions, which is also supported by −V(r)/G(r)<1 and low WBI and *δ* values. In the Fluoride, Chloride, Bromide and Hydroxide complexes, the value of H(r) is negative with 1.05≤−V(r)/G(r)≤1.31, indicating weak covalent natures of Pu-F^−^/Cl^−^/Br^−^/OH^−^ bonds. It is in particular important to note that there are more electron accumulations located at Pu–OH^−^ BCPs in PuO_2(OH_${)}_{4}^{2-}$ complexes relative to other Pu–ligand bonds, and values of H(r) are slightly larger in absolute values than for the other ligands. This indicates that the affinity between plutonyl (VI) and OH^−^ group is strongest among studied ligands. It is also worth noting that the interaction between Pu and Cl atoms in Plutonyl (VI) chloro complexes are significantly enhanced when the H_2_O ligands are coordinated, as illustrated by the H(r) and −V(r)/G(r) values in Table 4. The same conclusion can be drawn for the hydroxygen complexes based on H(r) and −V(r)/G(r) values. In PuO_2_Cl_2_(H_2_O)_2_, PuO_2_Cl_2_(H_2_O)_3_, PuO_2_Cl(H_2_O)_4_ and PuO_2_(OH)_2_(H_2_O)_3_ complexes, the Pu-L bonds shows predominant ionic character, but with some degree of covalency. Vallet *et al.* [13] recently found the U–$O_{L}$ bonds in UO_2_(O_2_)(H_2_O)_3_ and UO_2_(CO_3_)(H_2_O)_3_ complexes which have large *ρ* values and more negative H(r) values, are much stronger than other U–L bonds (L = F^−^, Cl^−^, OH^−^, H_2_O). However, from Table 4, one can see that Pu–$O_{L}$ interactions in studied Plutonyl (VI) peroxide and carbonate complexes are obviously weaker than those Pu–F^−^/Cl^−^/Br^−^, Pu–$O_{L}$ (L = ${\text{NO}}_{3}^{-}$) chemical bonds, which show typical closed-shell characteristics. Therefore, the ligand affinity of plutonyl(VI) is significantly different from Uranyl(VI).

### 3.4. Interaction Quantum Atom (IQA) Analyses

To interpret the physical nature of the Pu–L interatomic interaction and its local contribution to the overall energy of one Plutonyl (VI) complex, the computationally expensive but highly useful IQA energy decomposition scheme is considered. IQA defines the interaction $E_{int}^{AB}$ between two atoms as a competing contribution made by classical components (interaction energy between electrons and nuclei as well as coulombic interaction between electrons of atom A and B) conveniently combined as $V_{cl}^{AB}$, and quantum-mechanical contribution, as $V_{xc}^{AB}$. The calculated decomposition of Pu–L interaction energy in the studied Plutonyl (VI) complexes is collected in Table V, from which one can see that all Pu-L bonds except for Pu–N(${\text{NO}}_{3}^{-}$) and Pu–C(${\text{CO}}_{3}^{2-}$) shows negative $E_{int}^{AB}$ values, suggesting the stabilizing contribution. Among all Pu-L chemical bonds, the strong Pu–OH bond in PuO_2_(OH${)}_{4}^{2-}$ is characterized by the largest $|E_{int}^{AB}|$ value. This result is in line with QTAIM parameters and WBI values mentioned above. When the relative values of $V_{cl}^{AB}$ were considered, we found that all Pu–L bonds except for Pu–$N_{L}$(L = ${\text{NO}}_{3}^{-}$) and Pu–$C_{L}$(L = ${\text{CO}}_{3}^{2-}$) are predominately derived from the electrostatic attractive interaction. However, it is important to note that the $V_{xc}^{AB}$ values from quantum-exchange correlation are in the range of −38.91 to −81.58 kcal/mol. These quantities can not be ignored and also make a significant contribution to total interaction energy. Therefore, the quantum-exchange term is also important for the stabilizing of interaction. On the other hand, the Pu–N and Pu–C interactions are characterized by extremely large electrostatic repulsion with positive values of $V_{cl}^{Pu-N}=247.87$, $V_{cl}^{Pu-C}=596.13$ kcal/mol, respectively, which is responsible for ring critical point (RCP) in QTAIM. We also calculated the $V_{xc}^{AB}/E_{int}^{AB}$ ratio, conveniently called the exchange-interaction ratio (EIR). The $V_{xc}^{AB}$ term is always negative, therefore, when EIR>0, it can identify a locally stabilizing interatomic interaction, while locally destabilizing is characterized with EIR<0. From Table 5, one can see that the locally stabilizing contributions in Pu–OH_2_ and Pu–$O_{L}$(L=${\text{CO}}_{3}^{2-}$) are almost completely made by electrostatic attractive interactions. On the other hand, Pu–$N_{L}$(L=${\text{NO}}_{3}^{-}$) and Pu–$C_{L}$(L=${\text{CO}}_{3}^{2-}$) destabilizing interactions originate entirely from electrostatic repulsive term. For the rest of the Pu–Ligand bonds, the stabilizing interactions are characterized with dominant electrostatic term and non-ignorable quantum-exchange contribution.

### 3.5. Electron Localization Function (ELF) Analyses

We now consider the behavior of another density-based analytical tool, the electron localization function (ELF, denoted by η(r)). η(r) is a scalar field, and its topology can be analyzed in a manner similar to that of ρ(r) [38]. According to the ELF criterion, for covalent bonding, a typical maximum value of the ELF connecting two atoms is closer to 1.0, the higher the ELF value is, the stronger the bond is. The values of ELF associated with (3,−1) BCP between Pu and $O_{yl}$ and ligand are collected in Table 6, one can see that the ELF values corresponding to Pu–$O_{yl}$ bonds are significantly larger than those of Pu-ligand, suggesting the strong covalent nature of Pu–$O_{yl}$ bonds. These results correlate excellently with conclusions from QTAIM. Moreover, one can find the variation of ELF in these Pu–$O_{yl}$ bifurcation points is very small, indicating that different ligand fields do not significantly affect the Pu–$O_{yl}$ interaction—for the ELF values associated with Pu and ligands suggest the weak interaction between them. Comparing ELF values, one can obtain that Pu–OH^−^, Pu–F^−^, Pu–Cl^−^, Pu–Br^−^ bonds are slightly stronger than Pu–L bonds in rest complexes studied.

### 3.6. Noncovalent Interaction (NCI) Analyses

NCI index based on the relation between the electron density and reduced density gradient (RDG) was proposed by Yang *et al.* [18] to identify the weak interactions. The RDG is defined as follows:

(1) $$s\left(\rho \right)=\frac{1}{2{\left(3\pi^{2}\right)}^{1/3}}\frac{\left|\nabla \rho \right|}{\rho^{4/3}}$$

NCI resorts to the sign of the second eigenvalue of the Hessian ($\lambda_{2}$) of the electron density due to its sign dependence on different situations. More specifically, the $\lambda_{2}$ value will be negative in bonding cases and positive in most of the non-bonding repulsive situations. Generally, NCI procedure maps the product of the sign of the second eigenvalue of the Hessian, sign ($\lambda_{2}$) times the density onto an isosurface of RDG (*s*), accessing a pictorial display of the most relevant attractive (blue) and repulsion interaction (red) in a system. In addition, the 2-dimension (2D) scatter plots between sign($\lambda_{2}$)*ρ and RDG (*s*) can be used for NCI analyses. NCI allows for distinguishing which of the weak interactions are attractive or repulsive, and it can reveal those interactions that are not easily detected by QTAIM.

**Figure 4:**
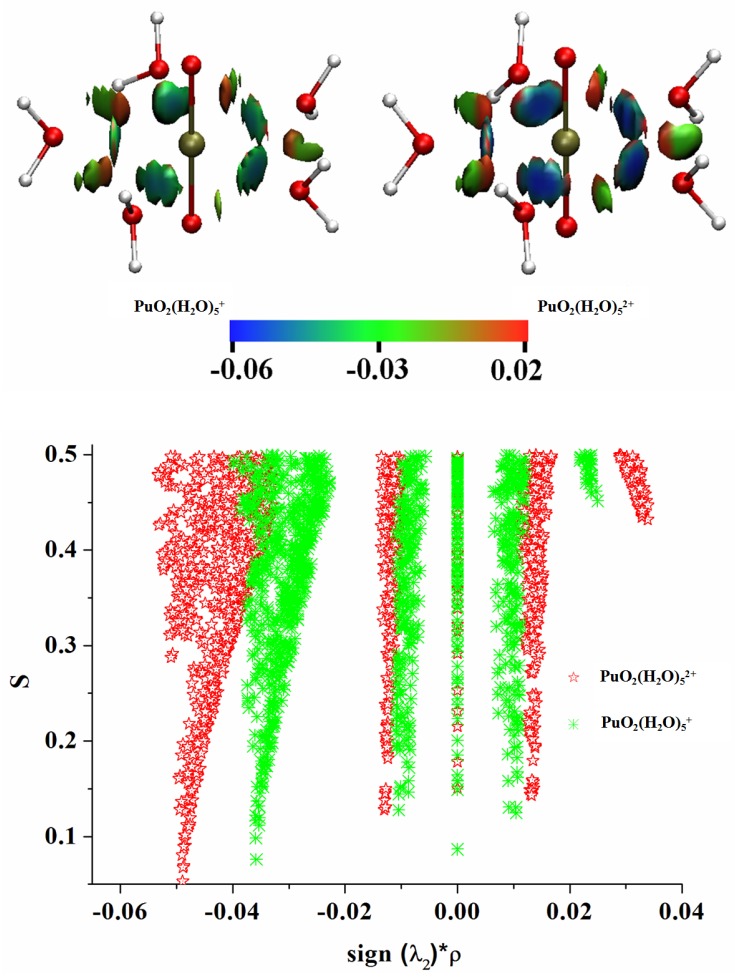
2D scatter plots of the RDG *vs.* sign($\lambda_{2}$)*ρ and corresponding 3D isosurfaces were generated with s=0.5 for PuO_2_(H_2_O${)}_{5}^{+}$ and PuO_2_(H_2_O${)}_{5}^{2+}$ aquo complexes.

**Figure 5:**
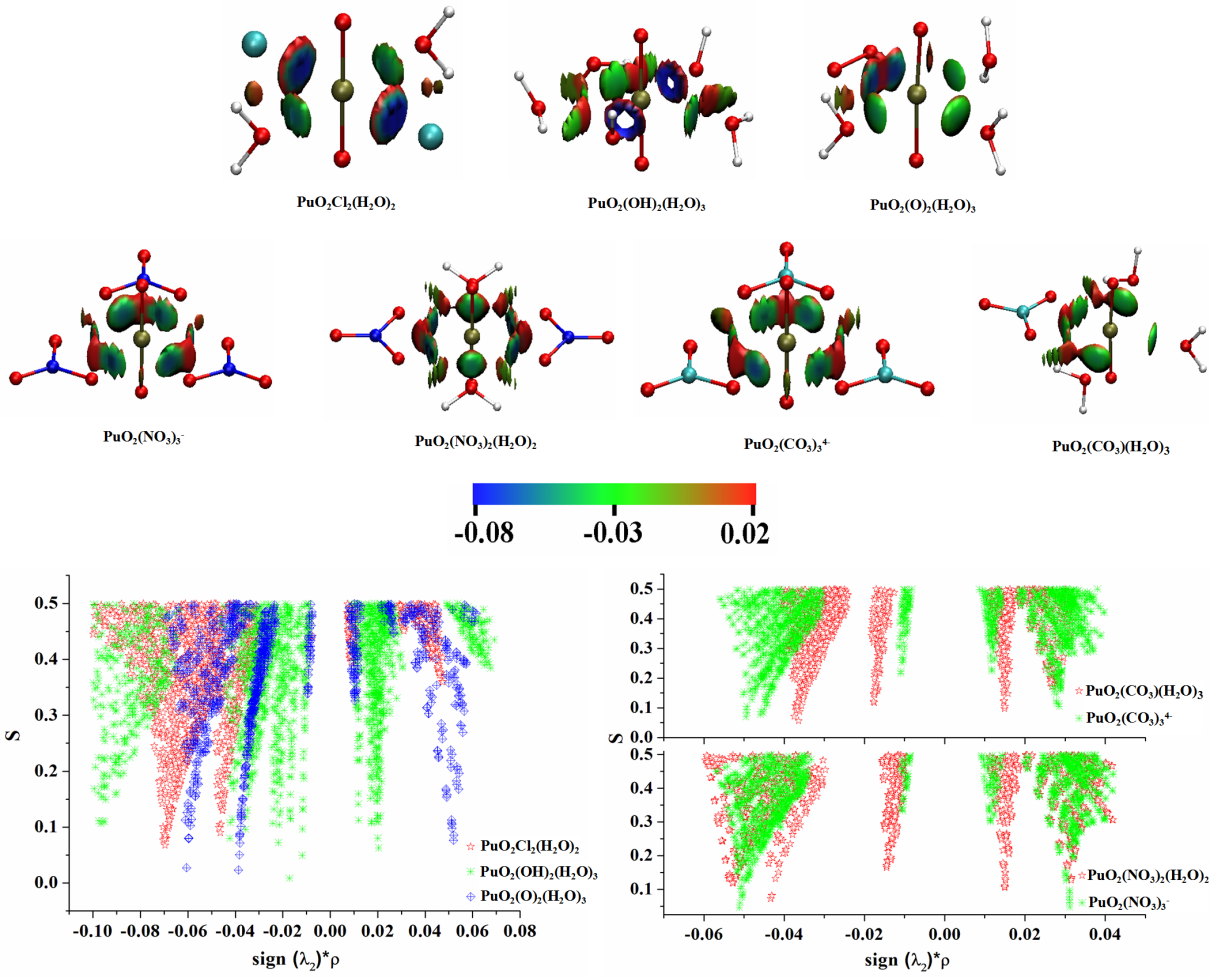
2D scatter plots of the RDG *vs.* sign($\lambda_{2}$)*ρ and corresponding 3D isosurfaces were generated with s=0.5 for Plutonyl (VI) complexes with Cl^−^, OH^−^, $O_{2}^{2-}$, ${\text{NO}}_{3}^{-}$ and ${\text{CO}}_{3}^{2-}$ ligands.

As we know that the preference for actinides to form complexes generally follows their effective ion charges, in the order of AnO_2_^2+^ > AnO_2_^+^, therefore, the PuO_2_(H_2_O${)}_{5}^{2+}$ (VI) complex was more favored in stability than PuO_2_(H_2_O${)}_{5}^{+}$ (V). As Table IV shows, the Pu–OH_2_ bonds in both PuO_2_(H_2_O${)}_{5}^{+}$ (V) and PuO_2_(H_2_O${)}_{5}^{2+}$ (VI) aquo complexes possess clear closed-shell characters. We can not however directly distinguish the Pu–OH_2_ interaction strengths in Plutonyl (V), Plutonyl (VI) aquo complexes from topological parameters (Table 4) within QTAIM framework. NCI isosurfaces of PuO_2_(H_2_O${)}_{5}^{+}$ (V) and PuO_2_(H_2_O${)}_{5}^{2+}$ (VI) are depicted in Figure 4, from which one can see that Pu-OH_2_ interaction in PuO_2_(H_2_O${)}_{5}^{+}$ (V) corresponding to light blue isosurface is slightly weaker than that in PuO_2_(H_2_O${)}_{5}^{2+}$ (VI) showing blue isosurface. The characteristic spike in 2D scatter plot, which corresponds to Pu–OH_2_ interaction, directed toward the sign ($\lambda_{2}$)*ρ value is −0.049 for PuO_2_(H_2_O${)}_{5}^{2+}$, and is much lower than that of PuO_2_(H_2_O${)}_{5}^{+}$ (−0.035). NCI analyses clearly indicate that the stronger Pu–OH_2_ interaction exists in PuO_2_(H_2_O${)}_{5}^{2+}$ (VI), which is responsible for its higher preference in stability. Apart from the bonding interaction between Pu and OH _2_, there are two gradient spikes in s(*ρ*) of Figure 4 (bottom), one is attractive interaction derived from the van der Waals interaction between neighboring H_2_O molecules (around −0.01 spikes), and the other corresponds to steric repulsion (around 0.01 spikes) between Pu and neighboring two H_2_O molecules. For other studied complexes with Cl^−^, OH^−^, $O_{2}^{2-}$, ${\text{CO}}_{3}^{2-}$ and ${\text{NO}}_{3}^{-}$ ligands, the NCI isosurfaces and corresponding 2D scatter plots are pictorially presented in Figure 5. Both 3D isosurfaces and 2D scatter graphs show that bonding interaction between Pu and OH^−^ which corresponds to low gradient spike of -0.10 is significantly stronger than Pu–Cl^−^ and Pu–$O_{2}^{2-}$ bonds, and the interaction strength sequence is ${\text{OH}}^{-}>{\text{Cl}}^{-}>O_{2}^{2-}$, in line with QTAIM and IQA results. For carbonate and nitrate complexes, the low gradient spike corresponding to Pu–O(NO_3_^−^) interaction in nitrate complexes is directed toward more negative sign($\lambda_{2}$)*ρ values relative to that in carbonate complexes, indicating the slightly stronger Pu–O(NO_3_^−^) bonding in nitrate complexes. We also note that there exist significant steric interactions between Pu and C/N atoms in both carbonate and nitrate complexes which have been revealed by IQA analyses, and this correlates very well with the QTAIM analyses where the bond path between Pu and C/N atoms are not observed. We also should mention that the Pu–OH_2_ interaction strengths, corresponding to the peaks from −0.01 to −0.05 are very different for studied complexes.

## 4. Conclusions

The geometric structures of plutonyl(VI) complexes in gaseous and aqueous phases were obtained with B3LYP functional in conjunction with relativistic effective core pseudopotential (RECP) and SARC basis set, respectively. The predicted geometry structures by all-electron SARC basis set are more accurate than RECP results. The consideration of solution effect results in the elongation of Pu–$O_{yl}$ bonds and the shortening of Pu–OH_2_ bonds for all studied aquo complexes. Moreover, the Pu–$O_{yl}$ and Pu–OH_2_ bond lengths are more close to the experimental values when solution effect is considered.

NPA analyses showed that there are significant electron promotions from 7s orbital to 6d and 7p orbitals when Pu atoms are involved in chemical bonds. The Pu–$O_{yl}$ chemical bonds, in which oxygen atoms accept fewer electrons, correspond to the stronger bond strength. The interesting linear dependence between Pu–$O_{yl}$ bond orders and the charge of oxygen atoms revealed the covalent characters of Pu–$O_{yl}$ bonds.

By employing the wavefunction obtained at B3LYP level of theory, we have investigated the binding natures of a series of plutonyl (VI) complexes utilizing QTAIM, IQA, ELF and NCI methods. Our QTAIM analyses indicate that the significant closed-shell interactions are suggested for Pu–Ligand bonds in almost all complexes studied. It is worth noting that the Pu–Ligand interaction shows a slight degree of covalency in some complexes. Moreover, the ligand affinity of plutonyl (VI) is different from uranyl(VI) analogies. IQA energy decomposition scheme was utilized to probe into the interaction between Pu and ligands, the electrostatic attractive interaction is characterized as the dominate stabilizing contribution, but the quantum-exchange term is also non-ignorable, making obvious contribution to total interaction energy. ELF was also employed to identify the bonding natures, and was found to draw similar conclusions to QTAIM.The NCI index results indicate that NCI is a successful extension of QTAIM. Some weak interactions, like Pu–OH_2_, H_2_O–OH_2_, which can not be distinguished in QTAIM, can be successfully identified with NCI. Moreover, the steric repulsion can be found with NCI analyses. We hope our electron density topological analyses are useful for a deep understanding of Pu–Ligand chemical bondings in plutonyl (VI) complexes.

## Figures and Tables

**Table 1:** The calculated bonding lengths (Å) of bare plutonyl (VI) and Plutonyl (VI) aquo species calculated at B3LYP/SARC-ZORA level of theory. The structural parameters calculated with B3LYP/SDD are given in the parentheses.

Species	Gas		Solution		Expt	
	Pu–O_*yl*_	Pu–OH_2_	Pu–O_*yl*_	Pu–OH_2_	Pu–O_*yl*_	Pu–OH_2_
${\mathrm{PuO}}_{2}^{2+}$	1.711(1.673)		1.716(1.723)			
PuO_2_(H_2_O${)}_{5}^{2+}$	1.751(1.703)	2.451(2.467)	1.763(1.716)	2.402(2.449)	1.74	2.41
${\mathrm{PuO}}_{2}^{+}$	1.748(1.714)		1.771(1.785)			
PuO_2_(H_2_O${)}_{5}^{+}$	1.808(1.766)	2.539(2.591)	1.832(1.779)	2.497(2.597)	1.81	2.47

**Table 2:** The calculated bonding lengths (Å) of a series of Plutonyl (VI) complexes in gaseous and aqueous phases calculated at B3LYP/SARC-ZORA level of theory, the structural parameters obtained with B3LYP/SDD are given in the parentheses.

Species	Gas			Solution		
	Pu–O_*yl*_	Pu–L	Pu–OH_2_	Pu–O_*yl*_	Pu–L	Pu–OH_2_
PuO_2_$F_{4}^{2-}$	1.811(1.773)	2.222(2.225)		1.806(1.766)	2.201(2.209)	
PuO_2_${\mathrm{Cl}}_{4}^{2-}$	1.772(1.742)	2.749(2.743)		1.775(1.737)	2.706(2.692)	
PuO_2_Cl_2_(H_2_O)_2_						
Cis-	1.768(1.729)	2.612(2.582)	2.445(2.513)	1.768(1.732)	2.671(2.664)	2.345(2.476)
Trans-	1.767(1.729)	2.623(2.591)	2.417(2.481)	1.768(1.728)	2.670(2.640)	2.349(2.421)
PuO_2_Cl_2_(H_2_O)_3_						
Cis-	1.767(1.725)	2.665(2.619)	2.574(2.624)	1.771(1.729)	2.738(2.680)	2.458(2.556)
Trans-	1.767(1.722)	2.717(2.661)	2.490(2.558)	1.771(1.728)	2.757(2.703)	2.441(2.523)
Expt				1.75	2.70	2.49
PuO_2_Cl(H_2_O${)}_{4}^{+}$	1.763(1.723)	2.617(2.566)	2.493(2.542)	1.768(1.719)	2.722(2.634)	2.424(2.486)
Expt				1.75	2.75	2.43
PuO_2_${\mathrm{Br}}_{4}^{2-}$	1.770(1.732)	2.907(2.904)		1.776(1.736)	2.853(2.862)	
PuO_2_(OH${)}_{4}^{2-}$	1.859(1.798)	2.281(2.288)		1.836(1.791)	2.243(2.272)	
PuO_2_(OH)_2_(H_2_O)_3_	1.836(1.742)	2.267(2.199)	2.548(2.611)	1.800(1.755)	2.193(2.201)	2.523(2.588)
PuO_2_(O)_2_(H_2_O)_3_	1.827(1.788)	2.398(2.379)	2.526(2.595)	1.838(1.796)	2.444(2.411)	2.471(2.563)
PuO_2_(NO_3_${)}_{3}^{-}$	1.762(1.729)	2.500(2.493)		1.761(1.737)	2.493(2.485)	
PuO_2_(NO_3_)_2_(H_2_O)_2_	1.767(1.722)	2.483(2.474)	2.466(2.524)	1.770(1.730)	2.512(2.490)	2.434(2.505)
Expt	1.727	2.432	2.497			
PuO_2_(CO_3_${)}_{3}^{4-}$	1.815(1.784)	2.558(2.544)		1.809(1.780)	2.467(2.469)	
PuO_2_(CO_3_)(H_2_O)_3_	1.814(1.773)	2.620(2.607)	2.514(2.581)	1.825(1.781)	2.678(2.673)	2.456(2.536)

**Table 3:** The Natural population analyses (NPA) charge (q) and electronic configurations (EC) of Plutonium, Oxygen in plutonyl (O_*yl*_) and coordinating atoms in ligands (L) of studied Plutonyl (VI) complexes, the Wiberg bond index (WBI) of Pu=O and Pu–L chemical bonds.

Species	*q*	*q*	*q*	*EC*	*EC*	*EC*	WBI	WBI
	(Pu)	(O_*yl*_)	(L)	(Pu)	(O_*yl*_)	(L)	(Pu=O)	(Pu–L)
${\mathrm{PuO}}_{2}^{+}$	2.04	−0.52	–	$5f^{5.31}6d^{0.86}$	$2s^{1.89}2p^{4.63}$	–	2.05	–
${\mathrm{PuO}}_{2}^{2+}$	2.50	−0.25	–	$5f^{4.87}6d^{0.93}$	$2s^{1.89}2p^{4.35}$	–	2.24	–
PuO_2_(H_2_O${)}_{5}^{+}$	1.44	−0.54	−0.89	$5f^{5.26}6d^{1.04}7p^{0.27}$	$2s^{1.81}2p^{4.72}$	$2s^{1.70}2p^{5.18}$	2.07	(0.24) ^*a*^
PuO_2_(H_2_O${)}_{5}^{2+}$	1.44	−0.26	−0.85	$5f^{5.05}6d^{1.26}7p^{0.31}$	$2s^{1.78}2p^{4.47}$	$2s^{1.67}2p^{5.17}$	2.29	(0.39)
PuO^2^$F_{4}^{2-}$	1.39	−0.46	−0.62	$5f^{5.03}6d^{1.29}7p^{0.29}$	$2s^{1.79}2p^{4.66}$	$2s^{1.90}2p^{5.71}$	2.10	0.65
PuO_2_${\mathrm{Cl}}_{4}^{2-}$	0.68	−0.39	−0.48	$5f^{5.19}6d^{1.56}7p^{0.44}$	$2s^{1.77}2p^{4.61}$	$3s^{1.89}3p^{5.59}$	2.18	0.86
PuO_2_Cl_2_(H_2_O)_2_								
Cis-	0.86	−0.35	−0.28	$5f^{5.20}6d^{1.51}7p^{0.39}$	$2s^{1.77}2p^{4.56}$	$3s^{1.87}3p^{5.40}$	2.23	1.14/(0.35)
Trans-	0.86	−0.35	−0.27	$5f^{5.23}6d^{1.48}7p^{0.39}$	$2s^{1.77}2p^{4.57}$	$3s^{1.87}3p^{5.39}$	2.22	1.14/(0.35)
PuO_2_Cl_2_(H_2_O)_3_								
Cis-	0.81	−0.34	−0.3	$5f^{5.18}6d^{1.50}7p^{0.42}$	$2s^{1.77}2p^{4.56}$	$3s^{1.87}3p^{5.43}$	2.23	1.08/(0.29)
Trans-	0.85	−0.34	−0.36	$5f^{5.16}6d^{1.48}7p^{0.43}$	$2s^{1.77}2p^{4.56}$	$3s^{1.88}3p^{5.48}$	2.23	1.01/(0.31)
PuO_2_Cl(H_2_O${)}_{4}^{+}$	1.09	−0.32	−0.18	$5f^{5.15}6d^{1.38}7p^{0.37}$	$2s^{1.78}2p^{4.54}$	$3s^{1.87}3p^{5.30}$	2.25	1.26/(0.32)
PuO_2_${\mathrm{Br}}_{4}^{2-}$	0.57	−0.40	−0.44	$5f^{5.20}6d^{1.62}7p^{0.46}$	$2s^{1.78}2p^{4.61}$	$4s^{1.89}4p^{5.55}$	2.18	0.89
PuO_2_(OH${)}_{4}^{2-}$	1.07	−0.51	−0.94	$5f^{5.15}6d^{1.36}7p^{0.34}$	$2s^{1.77}2p^{4.73}$	$2s^{1.76}2p^{5.17}$	2.08	0.79
PuO_2_(OH)_2_(H_2_O)_3_	1.13	−0.37	−0.86	$5f^{5.17}6d^{1.36}7p^{0.34}$	$2s^{1.77}2p^{4.59}$	$2s^{1.76}2p^{5.17}$	2.21	0.95/(0.26)
PuO_2_(O)_2_(H_2_O)_3_	1.31	−0.54	−0.31	$5f^{5.28}6d^{1.11}7p^{0.28}$	$2s^{1.81}2p^{4.73}$	$2s^{1.78}2p4.51$	2.07	0.40/(0.24)
PuO_2_(NO_3_${)}_{3}^{-}$	1.13	−0.32	−0.42	$5f^{5.09}6d^{1.31}7p^{0.39}$	$2s^{1.77}2p4.54$	$2s^{1.73}2p^{4.68}$	2.22	0.41
PuO_2_(NO_3_)_2_(H_2_O)_2_	1.15	−0.32	−0.44	$5f^{5.12}6d^{1.30}7p^{0.38}$	$2s^{1.77}2p^{4.54}$	$2s^{1.72}2p^{4.69}$	2.24	0.43/(0.32)
PuO_2_(CO_3_${)}_{3}^{4-}$	1.22	−0.52	−0.73	$5f^{5.10}6d^{1.20}7p^{0.35}$	$2s^{1.78}2p^{4.73}$	$2s^{1.71}2p^{5.01}$	2.06	0.41
PuO_2_(CO_3_)(H_2_O)_3_	1.40	−0.55	−0.68	$5f^{5.27}6d^{1.05}7p^{0.27}$	$2s^{1.81}2p^{4.74}$	$2s^{1.71}2p^{4.96}$	2.06	0.25/(0.25)

^*a*^ The Pu–OH_2_ bond orders are shown in parentheses.

**Table 4:** The topological parameters (a.u) for various studied complexes, the ligand is simply represented by L.

Pu-L BCPs					
Species	ρ	$\nabla^{2}\rho$	H(r)	δ(Pu,L)	−V(r)/G(r)
${\mathrm{PuO}}_{2}^{+}$	0.334	0.263	−0.322	2.181	1.90
${\mathrm{PuO}}_{2}^{2+}$	0.376	0.349	−0.393	2.374	1.88
PuO_2_(H_2_O${)}_{5}^{+}$	0.035	0.150	0.002	0.204	0.95
PuO_2_(H_2_O${)}_{5}^{2+}$	0.049	0.209	4.8 × $10^{-4}$	0.299	0.99
PuO_2_$F_{4}^{2-}$	0.081	0.363	−0.005	0.578	1.05
PuO_2_${\text{Cl}}_{4}^{2-}$	0.050	0.113	−0.008	0.577	1.21
PuO_2_Cl_2_(H_2_O)_2_				
Cis-	0.071 (0.045)	0.156 (0.184)	−0.016 (3.6 × $10^{-4}$)	0.822 (0.287)	1.30
Trans-	0.070 (0.047)	0.143 (0.204)	−0.016 (6.2 × $10^{-4}$)	0.823 (0.290)	1.31
PuO_2_Cl_2_(H_2_O)_3_				
Cis-	0.066 (0.036)	0.143 (0.131)	−0.014 (3.2 × $10^{-4}$)	0.742 (0.230)	1.28
Trans-	0.061 (0.045)	0.132 (0.181)	−0.012 (3.8 × $10^{-4}$)	0.673 (0.266)	1.26
PuO_2_Cl(H_2_O${)}_{4}^{+}$	0.074 (0.045)	0.156 (0.180)	-0.018 (2.1 × $10^{-4}$)	0.927 (0.281)	1.31
PuO_2_${\mathrm{Br}}_{4}^{2-}$	0.044	0.082	−0.007	0.589	1.25
PuO_2_(OH${)}_{4}^{2-}$	0.082	0.283	−0.011	0.666	1.14
PuO_2_(OH)_2_(H_2_O)_3_	0.105 (0.036)	0.360 (0.136)	−0.022 (3.5 × $10^{-4}$)	0.827 (0.213)	1.20
PuO_2_(O)_2_(H_2_O)_3_	0.061 (0.034)	0.241 (0.156)	−0.004 (0.002)	0.387 (0.211)	1.06
PuO_2_(NO_3_${)}_{3}^{-}$	0.051	0.185	−0.002	0.324	1.04
PuO_2_(NO_3_)_2_(H_2_O)_2_	0.054 (0.044)	0.188 (0.176)	−0.003 (6.0 $\times 10^{-4})$	0.349 (0.247)	1.05
PuO_2_(CO_3_${)}_{3}^{4-}$	0.050	0.158	−0.002	0.370	1.06
PuO_2_(CO_3_)(H_2_O)_3_	0.037 (0.037)	0.143 (0.155)	1.7 × $10^{-4}$ (8.1 × $10^{-4}$)	0.228 (0.240)	0.99

**Table 5:** Decomposition of Pu–L interaction energies within the IQA framework for all relevant interactions in studied Plutonyl (VI) complexes.

Complexes	Atoms	$V_{\mathrm{ne}}^{\mathrm{AB}}$	$V_{\mathrm{en}}^{\mathrm{AB}}$	$V_{\mathrm{nn}}^{\mathrm{AB}}$	$V_{c}^{\mathrm{AB}}$	$V_{\mathrm{cl}}^{\mathrm{AB}}$	$V_{\mathrm{xc}}^{\mathrm{AB}}$	$E_{\mathrm{int}}^{\mathrm{AB}}$	$V_{\mathrm{xc}}^{\mathrm{AB}}$/$E_{\mathrm{int}}^{\mathrm{AB}}$
		(a.u)	(a.u)	(a.u)	(a.u)	(kcal/mol)	(kcal/mol)	(kcal/mol)	
PuO_2_(H_2_O${)}_{5}^{2+}$	Pu–O(H_2_O)	−66.39	−53.69	58.35	61.09	−402.23	−39.53	−441.77	0.09
PuO_2_$F_{4}^{2-}$	Pu–F	−78.60	−66.90	72.76	72.27	−293.67	−74.05	−367.72	0.20
PuO_2_${\mathrm{Cl}}_{4}^{2-}$	Pu–Cl	−115.93	−103.53	111.48	107.66	−199.55	−59.61	−259.16	0.23
PuO_2_${\mathrm{Br}}_{4}^{2-}$	Pu–Br	−220.93	−201.90	216.87	205.69	−176.96	−56.48	−233.43	0.24
PuO_2_(OH${)}_{4}^{2-}$	Pu–O(OH^−^)	−71.53	−58.09	62.90	66.07	−412.27	−81.58	−493.85	0.17
PuO_2_(O)_2_(H_2_O)_3_	Pu–O($O_{2}^{2-}$)	−63.33	−56.27	60.51	58.90	−123.62	−46.44	−170.06	0.27
PuO_2_(NO_3_${)}_{3}^{-}$	Pu–O(${\text{NO}}_{3}^{-}$)	−61.93	−53.36	57.74	57.23	−198.92	−39.53	−238.45	0.17
	Pu–N(${\text{NO}}_{3}^{-}$)	−37.68	−39.63	42.89	34.82	247.87	−1.88	245.98	−0.008
PuO_2_(CO_3_${)}_{3}^{4-}$	Pu–O(${\text{CO}}_{3}^{2-}$)	−64.34	−51.98	56.21	59.50	−384.04	−38.91	−422.94	0.09
	Pu–C(${\text{CO}}_{3}^{2-}$)	−23.43	−33.31	36.03	21.66	596.13	−1.26	594.88	−0.002

**Table 6:** ELF η(r) values corresponding to (3,−1) BCPs associated with Plutonium center and $O_{yl}$ and Ligand in Plutonyl (VI) complexes studied.

Complexes	$\eta {\left(r\right)}_{\mathrm{Pu}-O_{\mathrm{yl}}}$	$\eta {\left(r\right)}_{\mathrm{Pu}-L}$
PuO_2_(H_2_O${)}_{5}^{2+}$	0.604	0.122
PuO_2_$F_{4}^{2-}$	0.579	0.172
PuO_2_${\text{Cl}}_{4}^{2-}$	0.593	0.229
PuO_2_${\text{Br}}_{4}^{2-}$	0.597	0.247
PuO_2_(OH${)}_{4}^{2-}$	0.567	0.227
PuO_2_(O)_2_(H_2_O)_3_	0.581	0.153
PuO_2_(NO_3_${)}_{3}^{-}$	0.576	0.166
PuO_2_(CO_3_${)}_{3}^{4-}$	0.594	0.152
